# Can digital storytelling improve health outcomes for immigrant and refugee populations? A scoping review

**DOI:** 10.1186/s12889-025-22209-1

**Published:** 2025-03-18

**Authors:** Sezer Kisa, Adnan Kisa

**Affiliations:** 1 Department of Nursing and Health Promotion, Faculty of Health Sciences, Oslo Metropolitan University, Oslo, Norway; 2 School of Health Sciences, Kristiania University of Applied Sciences, Oslo, Norway; 3 Department of International Health and Sustainable Development, Celia Scott Weatherhead School of Public Health and Tropical Medicine, Tulane University, New Orleans, La, United States of America

**Keywords:** Digital storytelling, Immigrant health, Refugee health, Health disparities, Cultural sensitivity

## Abstract

**Background:**

Digital storytelling (DST) combines narrative art with digital technology, and in doing so provides a medium for individuals, especially those within immigrant and refugee communities, to share their health experiences. While DST has been explored as a tool for improving health communication and literacy, its effectiveness remains uncertain due to methodological limitations in existing studies.

**Objectives:**

This review examined the use of DST in promoting health outcomes among immigrant and refugee communities, identified common challenges and best practices, and highlighted gaps in research and practice concerning DST’s use in these populations.

**Methods:**

The review used Arksey and O’Malley’s methodology to systematically explore the literature on DST’s effects on health outcomes in immigrant and refugee groups. It searched the MEDLINE/PubMed, Embase, Web of Science, PsychoInfo, and CINAHL databases for peer-reviewed research articles published in English up to February 15, 2025. The extracted data were categorized into four themes: DST and Health Outcomes, Challenges and Limitations, Best Practices and Effective Strategies, and Research and Practice Gaps.

**Results:**

DST has been applied in diverse healthcare contexts, including HPV vaccination promotion, chronic disease management (e.g., diabetes), and mental health interventions. Some studies reported improvements in health knowledge, self-care behaviors, and psychological well-being. However, the strength of evidence is limited, as most studies employed qualitative or quasi-experimental methods, relied on self-reported outcomes, and had small sample sizes. Challenges to DST implementation included technological barriers, digital literacy issues, feasibility concerns, and the need for culturally tailored interventions.

**Conclusions:**

DST shows promise as a culturally adaptable tool for health communication, but its effectiveness as a standardized health intervention remains unproven. Healthcare practitioners may consider DST as a complementary strategy for education and behavioral support in specific areas, such as vaccination campaigns and chronic disease management. However, further high-quality, controlled studies are necessary to evaluate its long-term impact, feasibility, and cost-effectiveness before widespread implementation. Future research should prioritize rigorous methodologies, objective outcome measures, and longitudinal assessments to establish DST’s role in public health interventions.

## Introduction

Storytelling has long been a fundamental tool for communication, education, and behavior change in healthcare [1–4]. Traditional storytelling methods have been used to share health knowledge, foster emotional connections, and encourage behavior change by presenting relatable narratives [1, 3]. Building on this foundation, digital storytelling (DST) incorporates digital media to enhance engagement and accessibility, expanding the potential applications of storytelling in healthcare [2, 4].

DST extends traditional storytelling by incorporating digital media, allowing for the creation of video clips that combine personal narratives with visual and audio elements [2, 3, 5]. It has also been defined as a creative process that involves visual, oral, and written communication [1]. DST was originally used in community, artistic, and therapeutic contexts, and has subsequently been applied in education, knowledge translation, health, community development, cultural preservation, and qualitative research. In a health context, DST offers a potential approach for individuals to share their health journeys by merging narrative practices with digital components such as text, images, audio narrations, background music, and videos [2, 4, 6]. This method allows one to hear the authentic voices of patients and facilitates a more engaging form of communication among healthcare users, which has been suggested to aid emotional healing for storytellers and increase empathy among viewers [3, 5, 7–9]. While DST has been explored for its potential to empower patients, improve healthcare education, foster emotional well-being, enhance self-knowledge, and promote professional identity, the extent of its effectiveness remains uncertain due to limited research and a lack of high-quality studies validating these outcomes [3, 5, 10]. Personal narratives may improve understanding and knowledge of health conditions, increase awareness of patients’ health-related experiences, provide emotional support, and influence behavioral changes and patient empowerment, but further research is needed to determine the impact of DST in diverse healthcare settings [10–13].

The adaptability of DST to different cultural and linguistic contexts makes it particularly valuable for engaging patients across diverse demographics [6, 14–19]. However, implementing DST in a healthcare setting has its challenges, including access to technology, digital literacy, resource demands, and potential emotional distress for storytellers. Ethical concerns include privacy risks, the potential for misrepresentation of personal narratives, unintended emotional harm from revisiting traumatic experiences, and the need for informed consent regarding the use and distribution of digital stories [6, 20]. There are also concerns about loss of privacy and oversimplification or misrepresentation due to cultural differences [6]. Using narrative runs the risk of having patients’ decisions influenced by personal experiences rather than statistical data [21]. Additionally, maintaining a project’s sustainability and evaluating its long-term impacts remain significant hurdles due to limited funding, lack of institutional support, and the need for ongoing training of facilitators. Other persistent barriers include technological constraints, such as access to reliable internet and digital devices, which disproportionately affect underserved communities. Language and cultural differences can also hinder the effectiveness of DST, as narratives may require careful translation and adaptation to maintain their intended meaning. Furthermore, participant engagement can be challenging, especially for individuals with limited digital literacy or those who may feel uncomfortable sharing personal health experiences in a digital format. These barriers highlight the need for tailored interventions that address accessibility, cultural relevance, and long-term feasibility [8, 9, 22–25]. Regardless of these challenges, DST remains a promising tool for promoting patient education, emotional well-being, and health behavior change, particularly in culturally and linguistically diverse settings [6]. DST has been applied in various healthcare contexts, demonstrating its potential to improve patient engagement, education, and emotional well-being. It has been used to reduce anxiety in cardiovascular patients by helping them share experiences and gain reassurance from others with similar conditions. In HIV/AIDS awareness campaigns, DST has facilitated the dissemination of culturally relevant health information, increased knowledge and reducing stigma among affected communities. Additionally, DST has played a role in chronic disease management, particularly for conditions like type 2 diabetes, by providing patients with relatable narratives that reinforce self-care behaviors and treatment adherence. By incorporating personal stories into health education, DST enhances comprehension, fosters emotional connections, and encourages behavior change, making it a valuable tool for improving health literacy and public health interventions [15, 16, 18, 19, 22].

Although DST has been considerably investigated, no published reviews or study protocols were found that directly map its use in immigrant and refugee populations. Because of the importance of cultural sensitivity in designing effective health interventions, this scoping review sought to identify existing research and practice gaps in employing DST with immigrant and refugee communities, particularly in relation to health promotion. It attempted to answer the following questions:

(1) How has DST been employed to improve health outcomes among immigrants and refugees?

(2) What challenges and limitations have practitioners and participants encountered in implementing DST within these groups?

(3) Which best practices and strategies have proven effective in employing DST in such contexts?

## Methodology

This scoping review integrated the methodologies that were described by Arksey and O’Malley [23] and further refined by Levac et al. [24]. It aimed to systematically map the literature on the impact of DST in immigrant and refugee communities. The research team consisted of two reviewers, who are also the authors of this work. One holds a PhD in Public Health, Policy, and Management, and the other in Global Health and Health Outcomes. Through in-person and virtual meetings, we formulated the overarching research question and defined the search terms, identified the databases for the literature search, established the inclusion and exclusion criteria, and devised methods for resolving any disagreements among the reviewers. We selected the MEDLINE/PubMed, Embase, Web of Science, PsychoInfo, and CINAHL databases due to their extensive coverage of medical, psychological, and health literature. Our search strategy was designed to identify studies on DST within healthcare contexts for immigrant and refugee populations while ensuring inclusivity of diverse terminologies used in the field. The search terms included (“digital stories” OR “digital storytelling” OR “digital narrative” OR “digital ethnography” OR “digital media” OR “online storytelling” OR “electronic storytelling” OR “web-based storytelling”) AND (“refugee” OR “migrant” OR “immigrant” OR “asylum seeker” OR “health”). The inclusion of “health” was intended to filter studies relevant to healthcare applications while maintaining flexibility in capturing various health-related disciplines. Given the interdisciplinary nature of DST research, we prioritized a balance between specificity and breadth to avoid inadvertently excluding relevant studies.

The search was first performed by one reviewer, and the selection processes were then conducted by two reviewers, who removed duplicates and separately screened titles and abstracts. The selected articles’ reference lists were hand-searched to find additional relevant studies. The full-text review and data extraction were also performed independently, with any disagreements resolved through discussion. Our review did not include a formal quality assessment of the included studies, in line with Arksey & O’Malley’s [23] recommendations for scoping reviews. We limited our review to peer-reviewed research articles published in English up to January 31, 2024. To ensure the timeliness and relevance of the information, we re-ran the search through February 15, 2025. While additional studies were published during this period, none met the inclusion criteria, and therefore, the original dataset remains unchanged. Studies were excluded if they did not focus on DST, were not related to immigrant or refugee populations, were not based on empirical research, or were published in languages other than English. Editorials, review studies, commentaries, and case studies were also excluded after reviewing their reference lists for additional studies because the focus was on original empirical data to systematically assess the implementation, impact, and effectiveness of DST interventions based on primary research rather than secondary interpretations or expert opinions. The leading reviewer finalized the selection process. The manuscripts were organized by using the reference management software EndNote (version 21).

Data extraction from each included study was done by each reviewer independently. The findings of this review were presented according to the Preferred Reporting Items for Systematic reviews and Meta-Analyses extension for Scoping Reviews (PRISMA-ScR) Checklist [25]. The characteristics of the studies were presented by author/year and country, study design, population, DST context, and study purpose (Table 1). The extracted data were categorized into four main themes: DST and Health Outcomes, addressing research question 1 (comprising health outcomes, description of DST interventions, key findings, and implications for practice (Table 2); Challenges and Limitations, responding to research question 2 (highlighting identified challenges, study limitations, and their impact on the DST process (Table 3); Best Practices and Effective Strategies, referencing research question 3 (identifying best practices, assessing the impact of effective strategies on the community (Table 4); and Research and Practice Gaps (identifying gaps, offering suggestions for future research (Table 5).

## Results

**Fig. 1:**
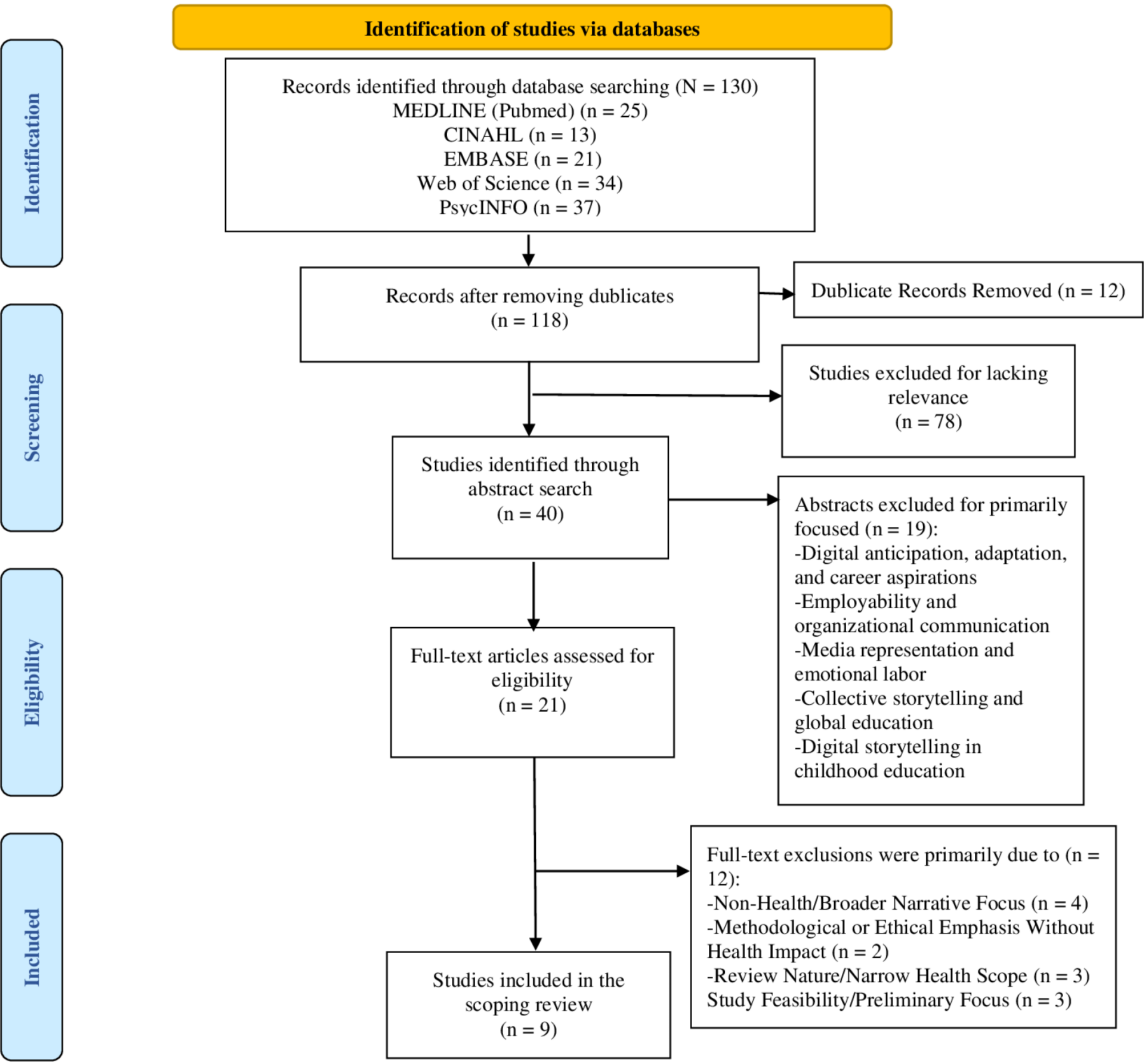
PRISMA

Although five databases were searched, together with the reference lists of the selected articles, only 122 useable articles were found. Of these, 20 full texts were reviewed, and 9 articles were included in the final review (Fig. 1).

### Study characteristics

The majority of the studies (seven) were conducted in the United States and only two in Australia. They covered both urban and community settings [7–9, 26–31], and the methods varied from mixed methods (two studies) and qualitative approaches (four) to quasi-experimental designs (two) [1–3, 10–13, 17, 18]. Three studies focused on the health outcomes of type 2 diabetes, one on reproductive health and social integration, one study about mental health and the psychological process of migration, and three studies focused on HPV vaccination (Table 1).

**Table 1:** Characteristics of the included studies

Author/Year, Country	Study Design	Population	DST* Context	Study Purpose
Lenette et al., 2015, Australia [22]	Qualitative, Ethnographic	Three African women from refugee backgrounds	Used for sharing personal narratives and facilitating collaborative ethnographic dialogue	To examine the use and effectiveness of DST in enhancing self-representation and agency among refugee women
Njeru et al., 2015, United States ADDIN EN.CITE [25]	Qualitative	24 Somali and 13 Latino immigrants and refugees	Development of a diabetes DST intervention	To develop a culturally appropriate DST intervention for improving type 2 diabetes (T2D) management among immigrants and refugees
Wieland et al., 2017, United States ADDIN EN.CITE [9]	Mixed-methods	25 adults (15 Latino, 10 Somali) with type 2 diabetes mellitus (T2DM)	DST intervention for immigrants and refugees with T2DM	To examine the effectiveness of DST in managing T2D among immigrants and refugees
McDonough & Colucci, 2019, Australia [24]	Qualitative	10 immigrants and refugees with mental health issues	Workshops under “Finding our way” project for creating digital stories on mental health recovery	To empower individuals and facilitate conversations on mental health, using personal digital stories for education and advocacy
Syed et al., 2022, United States [8]	Qualitative	26 Somali American emerging and older adults	Immigrant Stories Project: creation and analysis of digital stories	To explore the psychological process of migration for Somali refugees in the US through DST
Chen et al., 2022, United States [7]	Quasi-experimental, single-group, pretest-posttest	114 Vietnamese American mothers	HPV** vaccination promotion through personal stories	To co-develop and evaluate the effectiveness of a DST intervention aimed at increasing HPV vaccination intentions
Kim et al., 2023, United States ADDIN EN.CITE [31]	Mixed-methods	8 mothers (2 Vietnamese American, 6 Korean American) with vaccinated children	Workshops focusing on creating personal narratives related to HPV vaccination experiences	To assess the feasibility and acceptability of the DST intervention and to explore cultural experiences and attitudes towards HPV vaccination
Chen et al., 2023, United States [30]	Quasi-experimental, single-group study	164 mothers (50 Korean American, 114 Vietnamese American)	Employed to improve attitudes and intentions regarding HPV vaccination among mothers	To evaluate the feasibility, acceptability, and preliminary effects of a DST intervention on HPV vaccination rates
Lohr et al., 2023, United States ADDIN EN.CITE [23]	Two-group parallel randomized controlled trial	451 Hispanic/Latino individuals with T2D	Stories for Change (S4C) Diabetes DST intervention	To promote T2D self-management among Hispanic/Latino participants and evaluate the intervention’s efficacy

*DST: Digital Storytelling; **HPV: Human Papillomavirus

### DST and health outcomes

The studies showed the broad applicability of DST in health outcomes in applying diverse and tailored story-telling interventions to specific community needs. Several studies demonstrated DST’s efficacy in increasing HPV vaccination awareness and intentions among different immigrant mother groups [7, 26, 27]. Another study utilized DST to engage Korean American and Vietnamese American mothers, highlighting the importance of culturally and linguistically relevant storytelling in public health interventions [7, 26]. Two studies dealth with DST’s application to chronic disease management by revealing improvements in type 2 diabetes mellitus self-care behaviors [9, 29]. One study examined DST’s role in understanding the psychological impacts of migration among Somali refugees, thereby offering insights into mental health and social integration [8], while two studies highlighted DST’s impact on mental health recovery and community dialogue and emphasized its therapeutic potential [30, 31]. One study shed light on DST’s application in reproductive health and social integration among refugee women [28] (Table 2).

**Table 2:** DST and health outcomes

Author/Year	Health Outcomes	Description of DST Intervention	Key Findings	Implications for Practice
Lenette et al., 2015 [22]	Reproductive Health and Social Integration	DST used by refugee women to share experiences and challenges, employing digital media for personal narrative sharing	Enhanced understanding and empowerment, enabling women to articulate their life stories and challenges	Integrate DST in social work and health promotion to address complex health and social issues, amplifying marginalized voices for more inclusive care
Njeru et al., 2015 ADDIN EN.CITE [25]	T2D Management	Workshop involving Somali and Latino immigrants, focusing on creating personal stories about diabetes management	Stories reflected personal struggles and achievements, enhancing diabetes management engagement	Suggest utilizing DST as an engaging tool in chronic illness management among diverse immigrant groups
Wieland et al., 2017 ADDIN EN.CITE [9]	T2DM Self-Management	Culturally tailored DST with personal narratives for T2DM management among immigrants	High acceptance and improved confidence and behavioral intentions for T2DM management	Implement DST as a method for enhancing T2DM management, ensuring cultural tailoring for immigrant populations
McDonough & Colucci, 2019 [24]	Mental Health Recovery	Workshop for immigrants/refugees to create digital stories, combining personal narratives with digital technology	Process found to be empowering and safe, facilitating discussions on diversity, mental health, and recovery	Ensure DST projects are designed for psychological and cultural safety, used for education and advocacy, and include comprehensive evaluation and debriefing plans
Syed et al., 2022 [8]	Psychological Process of Migration	DST focusing on narratives of Somali refugees in the U.S.	Stories were mainly progressive or stable; highlighted different experiences between age groups	Use DST to allow immigrants and refugees to narrate their experiences and to inform targeted support and interventions
Chen et al., 2022 [7]	HPV Vaccination	Digital stories by Vietnamese American mothers, highlighting personal HPV vaccination narratives, available in English and Vietnamese	Mothers’ intention to vaccinate increased from 53 to 74%; high engagement and identification with stories	Leverage DST in health campaigns to boost vaccination rates, especially in underrepresented groups, ensuring cultural and linguistic relevance
Kim et al., 2023 ADDIN EN.CITE [31]	HPV Vaccination	Two-day DST workshops where mothers shared personal narratives and developed digital stories	Workshops were popular and well-received, promoting cultural sharing and influencing HPV vaccination perceptions	Utilize DST workshops to engage immigrant mothers in discussions about health interventions, potentially increasing HPV vaccination rates among their children
Chen et al., 2023 [30]	HPV Vaccination Rates	DST intervention featuring personal stories from Korean American and Vietnamese American mothers	Increased intention among mothers to vaccinate their children against HPV	Employ DST to address cultural and linguistic barriers, improving vaccination rates among immigrant populations
Lohr et al., 2023 ADDIN EN.CITE [23]	T2D Management	12-minute DST video and monthly texts focusing on diabetes self-management for Hispanic/Latino populations	High acceptability and motivation for self-management improvement reported	Apply DST methods for T2D management in Hispanic/Latino groups, incorporating regular follow-up communications

### The effectiveness of DST on health outcomes and implications for practice

The studies provided strong evidence of DST’s effectiveness in public health initiatives, particularly in vaccination campaigns and chronic disease management [7, 9, 26, 27, 29]. Other studies highlighted the potential of DST in mental health services and community health settings and advocated its use in psychological recovery and social reintegration [8, 30, 31]. Additionally, it was reported that DST significantly contributed to social work and health promotion, particularly in addressing complex social and health issues faced by refugees and marginalized groups [28] (Table 2).

### Challenges and limitations of DST

The studies reported several challenges and limitations to applying DST. Common challenges across diverse groups included scheduling conflicts, language barriers, and technological difficulties [27]. Specifically, first-generation immigrants and Vietnamese Americans encountered cultural and linguistic differences, compounded by participants’ time constraints [7, 26]. Challenges such as selection bias, nonrandom assignment, the varied characteristics of participants, limited participant information, differences in clinical practice, and issues with cross-sectional analysis were also reported in storytelling interventions [8, 9, 31]. The dynamics within storytelling workshops presented additional challenges, particularly when dealing with personal experiences and deeply distressing accounts [30]. Furthermore, maintaining motivation and engagement among different populations, time, resources, training requirements, and ethical concerns regarding privacy and narrative use were identified as significant challenges [28, 29] (Table 3).

**Table 3:** Challenges and limitations

Author/Year	Challenges Identified	Limitations of Study	Impact on DST Process
Lenette et al., 2015 [22]	Encountered logistical challenges such as time, resources, and training, alongside ethical concerns about privacy and narrative appropriation; mismatches between storytelling forms and cultural practices were also noted	The context-bound nature of the study and varying participant comfort levels along with sharing sensitive information could hinder broader application	Demonstrated DST’s potential for empowering self-representation while highlighting the necessity of ethical and cultural sensitivity in storytelling practices
Njeru et al., 2015 ADDIN EN.CITE [25]	Cultural and linguistic barriers were significant, along with participants’ physical discomfort and dietary management difficulties	The study’s scope was restricted to one community, limiting broader application, and it lacked concrete evidence of the intervention’s efficacy	Highlighted the critical role of culturally sensitive DST approaches and underscored the value of participatory processes in addressing these barriers
Wieland et al., 2017 ADDIN EN.CITE [9]	Faced nonrandom participant assignment, varied participant backgrounds, and differences in clinical practice, complicating the study’s execution	The design and variable measurement times may have introduced biases, alongside limited power for detecting significant differences, reducing its generalizability	These challenges suggest a need for careful DST design and implementation, particularly in culturally diverse healthcare settings, to enhance interpretability
McDonough & Colucci, 2019 [24]	Participants struggled with disclosing personal experiences and handling others’ distressing stories; challenges were also noted in group dynamics and script preparation	Emphasized the necessity of a safe environment and thorough preparation, with a need to balance risks and benefits for participants	The DST process was refined to foster a supportive, participatory environment, ensuring participant well-being and enhancing the storytelling’s depth and authenticity
Syed et al., 2022 [8]	Encountered selection bias and limited participant information, utilizing only a cross-sectional analysis approach	Faced with a small participant pool and reliance on outdated digital stories, the study’s results were hindered by potential selection bias and limited follow-up capabilities	The study highlights the importance of diverse participant selection and current context in DST, although limitations necessitate careful interpretation of outcomes
Chen et al., 2022 [7]	Vietnamese American mothers showed low initial engagement in the DST development process, with additional challenges posed by time constraints	The absence of a control group and ongoing data collection limited the study’s ability to make definitive conclusions; its findings may not extend beyond the specific community studied	Adjustments made to the DST process enhanced participant commitment and ensured cultural relevance, showcasing DST’s adaptability to participant needs
Kim et al., 2023 ADDIN EN.CITE [31]	Participants faced scheduling conflicts, language barriers, and technological issues, affecting their ability to contribute and engage	The study was conducted with a small, homogeneous group, limiting its diversity and generalizability	Despite these issues, modifications to the storytelling process increased participant engagement and allowed for greater cultural expression, indicating the adaptability of DST
Chen et al., 2023 [30]	Difficulty engaging first-generation immigrants due to significant language and cultural barriers, compounded by time constraints for participation	The study utilized convenience sampling, relied on a modest sample size, and lacked longitudinal follow-up and a control group	Tailoring of the DST intervention helped overcome language and cultural challenges, highlighting its potential for effective health communication in diverse populations
Lohr et al., 2023 ADDIN EN.CITE [23]	Challenges included engaging a diverse Hispanic/Latino population and maintaining motivation and engagement throughout the intervention	Predominantly female participants and the specific CBPR approach may limit the findings’ applicability to wider populations and settings	The findings call for strategies to ensure broader representation and inclusivity in DST, emphasizing the need for culturally tailored engagement methods

### Best practices and effective strategies

Use of inclusive language, respect for cultural differences, and engaging storytelling were identified as the best practices in enhancing DST [27]. Studies have applied cultural and linguistic tailoring, participatory approaches, and community engagement to DST interventions, with variations in reported effectiveness across different contexts. Culturally and linguistically congruent DST, along with sharing cultural norms and using native languages, were emphasized for effective communication and storytelling [7, 26]. The importance of utilizing DST for psychological analysis and creating culturally relevant narratives was highlighted [8]. Community-based participatory research (CBPR), cultural and linguistic tailoring, and the integration of personal narratives in DST further strengthened the story [9, 31]. Creating an inclusive and supportive environment, accommodating participant needs, and employing a participatory and democratic approach were crucial for successful DST workshops [30]. Emphasizing community involvement and maintaining cultural and linguistic relevance were also considered best practices for engaging diverse populations [29]. Lastly, using DST to enhance self-representation, agency, and the creation of counter-narratives supported individual and community empowerment [28] (Table 4).

Several effective strategies included the use of personal narratives, group environments for sharing experiences, and ensuring language accessibility, as these have been instrumental in engaging communities and addressing health issues [27]. Tailoring stories to match participants’ concerns, creating emotional resonance, and making interventions accessible remotely were strategies that increased engagement and relevance within communities [7, 26]. Collaborative and participatory research methodologies allowed participants to narrate their own experiences, thus enhancing the authenticity and impact of DST [8]. DST workshops and narrative development, focusing on managing chronic diseases like type 2 diabetes mellitus, demonstrated the power of shared experiences [9]. Collaborating with the community, using personal stories for motivation and education, and training community members in DST techniques improved health management and community engagement [31]. Tailoring workshop design to participant needs, incorporating multimedia elements, and ensuring psychological and cultural safety addressed various participant concerns, thereby enhancing the DST experience [30]. Finally, two studies called for employing bilingual and bicultural staff and involving the community in the storytelling process to help address health disparities and improve management practices [28, 29] (Table 4).

**Table 4:** Best practices and effective strategies

Author/Year	Best Practices	Effective Strategies	Impact on Community
Lenette et al., 2015 [22]	Utilizing DST for improved self-representation and agency; applying a participatory, strengths-based approach	Facilitating the collaborative creation of digital stories, including scriptwriting, voiceovers, and the use of personal images and music, followed by iterative feedback	Empowered individual storytellers, improved community understanding, and fostered stronger cultural and familial connections, enhancing social integration
Njeru et al., 2015 ADDIN EN.CITE [25]	Embracing an inclusive, culturally sensitive approach through CBPR* and integrating personal narratives effectively	Encouraging community collaboration, utilizing personal stories for educational purposes, and providing DST training to community members	Increased diabetes self-management skills and community engagement, leading to a strengthened sense of empowerment among participants
Wieland et al., 2017 ADDIN EN.CITE [9]	Implementing CBPR approaches, ensuring cultural and linguistic appropriateness, and maintaining engaging narrative content	Conducting DST workshops focused on T2DM, with stories developed by community members themselves	Improved self-management of T2DM among immigrant communities, showing potential for replication in similar contexts
McDonough & Colucci, 2019 [24]	Creating a supportive and inclusive environment in workshops, focusing on the needs and safety of participants	Tailoring the workshop structure to accommodate participant needs, integrating various multimedia elements, and ensuring a safe space for sharing personal stories	Enhanced mental health awareness and support within immigrant and refugee communities, promoting solidarity, and improving communal mental health literacy
Syed et al., 2022 [8]	Leveraging DST for in-depth psychological analysis and culturally relevant narrative creation	Using collaborative research approaches, enabling participants to express their migration stories and challenges	Provided Somali immigrants and refugees a platform to articulate their experiences, enhancing community understanding and integration
Chen et al., 2022 [7]	Development of storytelling interventions that are culturally congruent and linguistically appropriate	Creating DST pieces within a community-based participatory framework, directly involving the targeted community in content creation	Effectively addressed HPV-related health disparities in the Vietnamese American community, increasing awareness and preventive measures
Kim et al., 2023 ADDIN EN.CITE [31]	Use of inclusive language and cultural respect; engaging and relatable storytelling techniques	Implementing personal narrative workshops, creating supportive group environments, and ensuring content is accessible in multiple languages	Improved community engagement and awareness on HPV vaccination, leading to increased vaccination rates among Vietnamese American and Korean American communities
Chen et al., 2023 [30]	Adoption of culturally and linguistically tailored DST; reinforcing shared cultural norms and utilizing native languages	Customizing digital narratives to address specific cultural concerns, employing emotional resonance, and providing remote access to interventions	Significantly increased HPV vaccination intent among Korean American and Vietnamese American mothers, demonstrating effective cross-cultural communication
Lohr et al., 2023 ADDIN EN.CITE [23]	Applying CBPR principles, emphasizing cultural and linguistic engagement, and fostering active community participation	Utilizing personal storytelling in digital formats and employing bilingual and bicultural staff for more authentic engagement and data collection	Led to an improvement in T2D management and community health engagement, helping to reduce health disparities within the community

*CBPR Community based participatory research

### Limitations of the studies and identified gaps

Several limitations affecting the studies’ outcomes and generalizability were acknowledged. Small sample sizes, minimal diversity among participants, and the absence of longitudinal assessments were reported [7, 26, 27]. The reliance on previously collected stories and potential selection bias, along with limited follow-up opportunities, highlighted challenges in interpreting DST research [8]. The use of matched case-control designs and nonrandomized designs, along with variable measurement times, limited the interpretability and generalizability of the results [9]. The arbitrary number of created stories and the studies’ focus on single communities limited the evidence for intervention efficacy [31]. Safety, immersion, and participant preparation within workshops required careful consideration to mitigate risks and ensure beneficial participation [30]. Additionally, gender imbalance and the unique aspects of CBPR partnerships affected the replicability and generalizability of the findings [29]. Finally, the context-specific nature of the studies and variability in participants’ comfort and readiness to share sensitive stories highlighted the challenges in generalizing DST interventions [28] (Table 5).

The lack of studies focusing on culturally and linguistically diverse groups, especially regarding engagement and development of DST interventions [7, 26], non-randomized designs, limited generalizability, and variable measurement times exposed significant research gaps [9]. Furthermore, the limited scope of research regarding the generalizability of findings, arbitrary stories [31], the absence of formal evaluation and exploration of process considerations [30], research specifically addressing DST’s impact on male populations, and diverse CBPR required attention [29]. Lastly, there was a need for broader application and understanding of DST’s long-term impacts, especially among marginalized communities [28] (Table 5).

**Table 5:** Research and practice gaps

Author/ Year	Identified Gaps	Suggestions for Future Research	Implications for Practice
Lenette et al., 2015 [22]	Limited DST application in diverse social work environments and understanding of its long-term effects on marginalized groups	Undertake comprehensive studies that compare DST with traditional engagement methods and assess long-term community impacts	Encourage DST integration into social work, with specialized training for practitioners and guidelines emphasizing ethical storytelling practices
Njeru et al., 2015 ADDIN EN.CITE [25]	Generalizability issues, undefined optimal story quantity, and lack of proven efficacy	Define the most effective number of stories for impact; rigorously test the efficacy in varied community settings	Craft community-specific interventions and enhance DST skills among community health workers to address disparities effectively
Wieland et al., 2017 ADDIN EN.CITE [9]	Challenges with non-randomized design and limited scope of study applicability	Conduct larger, randomized studies across varied demographic settings with standardized metrics for clearer comparisons	Adapt DST interventions to fit specific cultural contexts and integrate them into standard diabetes care practices
McDonough & Colucci, 2019 [24]	Absence of formal evaluation and detailed exploration of DST processes for mental health in immigrant/refugee groups	Conduct detailed evaluations of DST projects, focusing on mental health impacts and processes in marginalized communities	Improve project designs and support mechanisms in mental health DST initiatives, ensuring diverse and inclusive narrative representation
Syed et al., 2022 [8]	Constraints due to small sample size, existing story reliance, and selection bias	Encourage larger, more inclusive studies with varied design approaches to capture a broader range of narratives	Revise DST approaches to minimize biases and better reflect the diverse experiences within immigrant and refugee groups
Chen et al., 2022 [7]	Inadequate involvement of Vietnamese American mothers in DST development	Broaden research to diverse groups and introduce control groups to enhance comparative understanding	Prioritize cultural and linguistic customization in health interventions to better serve diverse communities
Kim et al., 2023 ADDIN EN.CITE [31]	Limited research on DST’s effect on HPV vaccination among Vietnamese American and Korean American mothers	Investigate how DST influences cultural perspectives and vaccine attitudes more deeply in these communities	Implement DST to specifically target vaccination disparities, enhancing engagement and understanding in these communities
Chen et al., 2023 [30]	Scarcity of DST studies in diverse linguistic groups for HPV awareness	Examine secure, scalable DST platforms for better delivery and tracking, considering demographic variables such as children’s sex and generational differences in vaccine attitudes	Integrate culturally relevant DST as a standard tool in healthcare settings to promote HPV vaccination among diverse populations
Lohr et al., 2023 ADDIN EN.CITE [23]	Omitted specific research gaps	Expand research to assess the impact of DST on male populations and explore the efficacy of various CBPR approaches	Develop and implement DST programs that cater to the broader needs of Hispanic/Latino populations for effective T2D management

*CBPR: Community based participatory research

### Suggestions for future research

In the studies reviewed, it was stressed that future research should be designed with larger sample sizes, incorporate longitudinal designs, and ensure greater inclusivity and diversity when engaging different generations of immigrants in storytelling [8]. Investigating the optimal number of stories and testing intervention efficacy to provide valuable insights into DST effectiveness [31] is needed, along with in-depth exploration of DST’s impact [30]. Further research on DST’s effects on different demographics and in various CBPR contexts was also suggested [28, 29] (Table 5).

## Discussion

By being the first to focus on DST in addressing health disparities within immigrant and refugee communities, our study contributes to the understanding of how DST has been applied in health interventions. While DST has been explored as a culturally relevant and flexible tool in healthcare, the limited number of studies and variations in research designs make it difficult to establish its effectiveness with certainty. However, previous research suggests that DST may support self-reflection, facilitate understanding, boost confidence, strengthen social connections, and provide individuals with insights into their lived experiences [6, 10, 11, 32–34]. Further investigation is needed to better understand the specific mechanisms through which DST may influence health outcomes.

Socially, DST fosters a sense of belonging and shared experience, which may help reduce isolation and strengthen community ties [22]. Psychologically, storytelling provides a structured way for individuals to process complex emotions, reframe traumatic experiences, and develop a sense of agency over their narratives [8]. Emotionally, creating and sharing personal stories can be cathartic, offering storytellers validation and empowerment [24]. From a behavioral perspective, DST has been explored as a tool to engage audiences through relatable, real-life narratives, with some evidence suggesting it may enhance motivation for health-related actions [7]. However, due to the variability in methodologies and the lack of large-scale trials, further research is needed to determine how these mechanisms translate into measurable health improvements. Understanding these mechanisms is particularly valuable for those designing and implementing health interventions, as it helps identify the conditions under which DST may be most effective in promoting health-related behavior change.

Other studies on DST as an intervention for various populations align with our findings [35, 36]. A study on older adults found that the most common effects of DST included the promotion of mental health, an increased amount of meaningful community connections, greater digital literacy, the mitigation of negative ageism, and enhanced intellectual ability [32]. Another study about the reduction of pre-surgical anxiety highlighted DST’s broad applicability in alleviating health-related anxieties [18]. This consistency across studies underscores the versatility of DST as a therapeutic tool that transcends cultural and demographic boundaries. By promoting a narrative understanding, DST allows individuals to process their experiences, normalize their feelings, provide reassurance, and foster a community of shared experiences, thus reducing anxiety and other stress-related health conditions.

Health education means providing the knowledge, attitudes, and skills to enhance or maintain health, particularly within marginalized communities. One of the results of this review is DST’s effectiveness in health education. This result is consistent with the findings of Ezegbe et al. on the use of DST to enhance knowledge and risk perception of HIV/AIDS among Nigerian schoolchildren [37], and a study about health promotion and cancer awareness among rural Alaskan communities [38]. The results of a nurse-led project involving DST among hypertensive rural residents showed that the majority of the listeners had no unplanned provider visits, had no emergency department room visits, and were not hospitalized [39]. Another study showed how digital video storytelling overcame barriers to improved health outcomes [40]. By presenting health information through relatable stories, DST transcends traditional educational barriers, making complex information more accessible and memorable. This approach not only enhances understanding but also empowers individuals to make informed health decisions.

It has been well established in the literature that to improve health outcomes, patient education must be responsive to the individual’s needs [41]. Similar to the narratives emerging from our scoping review, DST can engage individuals in their health management and allow for interventions that address the specific cultural and contextual needs of the audience. Personalized storytelling can significantly enhance personal motivation and self-efficacy, leading to more sustained behavioral changes. The study by Kim et al. on smoking cessation among women living with HIV provides a compelling example of DST’s capacity for initiating and maintaining adjustments to health behavior [17]. Similarly, a study about the role of empathy within DST interventions, particularly among men who were seeking counseling for depression, align with our review’s insights on the power of relatable storytelling [16]. Creating narratives that resonate on a personal level can encourage a person to seek help and engage with health services. This empathetic approach in storytelling aids in breaking down stigmas and fostering a more compassionate understanding of health issues, crucial for engaging hard-to-reach populations. The innovative application of DST in legacy-making for children with cancer, as explored by Akard et al., opens new avenues for DST beyond traditional health education [15]. This suggests DST’s potential in addressing sensitive topics and supporting emotional well-being during challenging life events. Aligning with our review’s call for diverse applications, this underscores the need for culturally sensitive and contextually appropriate storytelling, ensuring that DST interventions are respectful, relevant, and resonant with the target audience’s experiences and needs. Our review found that inclusive language use and narratives tailored to specific community contexts are important strategies in applying DST. Inclusive language is defined by Likis as “respectful, accurate, unbiased, and consistent with the preferences of the individuals and communities who are being discussed” [42]. Likis goes on to state that language is powerful, and inclusive language which fosters respect promotes equity. When considering immigrant populations, these results should be considered by healthcare professionals who work with diverse populations.

Despite the promising outcomes associated with DST, both our scoping review as well as the literature identified several challenges related to the applications of DST. These include technological accessibility, maintaining participant engagement, and the development of culturally tailored interventions [19]. The studies highlight the need for ongoing support and dedicated resources to ensure the successful implementation and sustainability of DST projects, especially in communities with limited access to technology and healthcare. In conclusion, this integrated discussion emphasizes the significant yet untapped potential of DST in public health, particularly for immigrant, refugee, and other marginalized populations. Future research should address existing challenges, evaluate DST’s feasibility across different populations, and apply controlled trials or longitudinal studies to assess its long-term impact on health behaviors and outcomes.

### Limitations of the study

This scoping review has several limitations. Firstly, the studies demonstrated considerable variation in methodological rigor. While qualitative studies provided rich narrative data on participants’ experiences with DST, their findings were inherently limited by small sample sizes and a lack of generalizability. The quasi-experimental studies aimed to assess DST’s impact on health behaviors; however, they often lacked control groups, making it difficult to draw strong causal inferences. Mixed-methods studies offered valuable insights by combining qualitative depth with quantitative measures, yet they were constrained by small-scale implementations and self-reported outcomes. Since many DST studies rely on self-reported measures, there is a potential for response bias, social desirability bias, or recall bias, which may affect the accuracy of reported behavioral or health changes. Future research should incorporate objective measures, such as clinical health indicators or behavioral tracking, where feasible, to strengthen the evidence base. Additionally, none of the studies employed a large-scale randomized controlled trial (RCT), which is considered the gold standard for assessing intervention effectiveness. Given these methodological limitations, the findings should be interpreted with caution. While there is emerging evidence supporting DST’s role in health promotion, particularly in vaccination awareness and chronic disease management, the strength of this evidence is constrained by the predominance of exploratory and feasibility studies. Future research should incorporate more robust study designs, including larger sample sizes, controlled trials, and long-term follow-ups, to establish a clearer understanding of DST’s impact on immigrant and refugee health outcomes. Secondly, there may be selection bias due to the focus on specific health contexts of immigrants and refugees, potentially limiting the generalizability of findings to broader public health initiatives. Thirdly, the considerable variance in methodologies, designs, and populations among the included studies complicates result synthesis and comparison, which may affect their generalizability and interpretability across different settings and groups. Fourthly, the review only includes studies published in English and primarily focuses on peer-reviewed research articles. This approach may exclude valuable grey literature and other forms of scholarly output, which could lead to a limited understanding of the topic. The restriction to English may omit significant research from non-English speaking countries or communities, limiting the cultural diversity and depth of findings. Finally, the dynamic and evolving nature of DST and its technology means that recent innovations and practices might not be fully represented, leading to a potential underrepresentation of current practices and advancements in the field.

## Conclusion

In this review, we aimed to identify research and practice gaps that employed DST with immigrant and refugee communities, particularly in relation to health promotion. This review highlighted the potential adaptability of DST in promoting health outcomes among immigrants and refugees. It revealed that DST may serve as a bridge to overcome cultural and linguistic barriers to enhance health communication, foster community engagement, and empower individuals. The narratives created through DST provide a platform for individuals to share their unique health experiences, thereby facilitating more understanding and empathy among healthcare providers and peers. Despite the challenges identified, such as technological barriers and the need for cultural sensitivity, DST has been explored as a potential tool for transforming public health practices, particularly within diverse populations. By developing culturally sensitive content and respecting diverse backgrounds, DST could effectively address specific health concerns among immigrant and refugee populations.

Several suggestions emerged from this review. Future initiatives should focus on improving digital access and literacy within these communities. Moreover, there is a need for longitudinal research to assess the long-term impacts of DST on health behaviors and outcomes. Expanding the demographic focus to a wider range of immigrant and refugee groups will further enrich the DST literature and practice, providing a deeper understanding of how different populations interact with and benefit from DST. Addressing these gaps will not only enhance the potential effectiveness of DST interventions, it will also contribute to a more inclusive public health strategy that acknowledges and values the diversity of experiences within immigrant and refugee communities.

## Data Availability

No datasets were generated or analysed during the current study.
